# Understanding Preconception Women’s Needs and Preferences for Digital Health Resources: Qualitative Study

**DOI:** 10.2196/39280

**Published:** 2022-08-05

**Authors:** Ruth Elizabeth Walker, Sara Quong, Patrick Olivier, Ling Wu, Jue Xie, Jacqueline Boyle

**Affiliations:** 1 Monash Centre for Health Research and Implementation Monash University Clayton Australia; 2 Action Lab Monash University Clayton Australia; 3 Eastern Health Clinical School Monash University Box Hill Australia

**Keywords:** digital health, preconception, health promotion, behavior change, women's health, maternal health, digital health resource, healthy life style, qualitative analysis, online health information

## Abstract

**Background:**

Improving preconception health can benefit all women, their children, and their families regardless of their individual pregnancy intentions. Rapidly increasing access to information technology and online engagement have created opportunities to use digital health resources to engage with preconception women regarding lifestyle behaviors.

**Objective:**

This study explores how preconception women engage with digital health resources and online platforms to inform the design and development of a digital health resource to support women to make positive behavior change for their preconception health.

**Methods:**

This codesign research followed the Double Diamond process, which focuses on contextualization and explorative processes in phase 1 and ideation and development processes in phase 2. Phase 1 is reported on in this study and was undertaken via a series of 1-on-1 in-depth interviews with female participants (N=12) aged 18-45 years over 3 months. Interviews were designed to explore participants’ lived experiences in relation to their health and desired supports for healthy lifestyle behaviors. The first interview focused on participants’ perceptions of health and health behaviors, the second interview focused on social connections for health, and the third interview focused on digital health information and supports. Conversations from the first interview informed the development of the second interview, and conversations from the second interview informed the development of the third interview. Community advisors (N=8) met to provide feedback and advice to the researchers throughout the interview process. Qualitative analyses of transcripts from interviews were undertaken by 2 researchers before a deductive process identified themes mapped to the capability, opportunity, motivation, and behavior (COM-B) framework.

**Results:**

In total, 9 themes and 8 subthemes were identified from 124 codes. In relation to digital health resources, specifically, participants were already engaging with a range of digital health resources and had high expectations of these. Digital health resources needed to be easy to access, make women’s busy lives easier, be evidence based, and be reputable. Social connectedness was also highly important to our participants, with information and advice from peers with similar experiences being preferred over yet more online health information. Online communities facilitated these social interactions. Participants were open to the idea of chatbots and virtual assistants but acknowledged that they would not replace authentic social interactions.

**Conclusions:**

Codesigned digital health resources should be evidence based, reputable, and easy to access. Social connections were considered highly important to women, and designers of digital health resources should consider how they can increase opportunities for women to connect and learn from each other to promote health behaviors.

## Introduction

Optimal preconception health has benefits beyond fertility and pregnancy for mothers, children, and women who do not have children [1-3]. A public health view of the preconception period encompasses the months and even years prior to a potential pregnancy, when health behaviors may impact maternal health status at the critical weeks around conception [2,4]. Taking this broad view is useful when considering that the event of pregnancy is never certain, that a relatively large proportion of pregnancies are unplanned [5], and that some women remain nulliparous. Health behaviors in relation to nutrition, physical activity, and weight management can improve preconception health, prevent chronic disease across the life span [6-8], and assist with managing existing health conditions [9]. Positive health behaviors are often observed by others and can have a positive influence on children, families, and those in the broader community [10].

Women’s awareness of the importance of their health behaviors during the preconception period is low [11]. This issue is exacerbated by social, economic, and physical environments that tend to promote or enable suboptimal health behaviors [12]. Globally, the most rapid increase in rates of overweight and obesity is in reproductive aged women [13,14], and approximately 40% of women conceive with a BMI that is considered unhealthy [15]. Preconception behavior change interventions tend to target women of a reproductive age who are planning a pregnancy [16]. These women have been labeled pregnancy “intenders” [17]. Intenders are identified by their goal to conceive and motivation to have a healthy pregnancy [17,18]. Women of a reproductive age who are not planning a pregnancy in the foreseeable future have been labeled “nonintenders” [17]. Their health behaviors are equally important as intenders’ as they may eventually impact a future pregnancy, planned or unplanned, and the health of the women themselves [2,4]. Nonintenders are generally not motivated by the event or chance of a pregnancy, possibly far off in the future, to change health behaviors [17]. In fact, they tend not to resonate with the term “preconception” at all, considering it to be something that applies to those planning a pregnancy [19]. These women may have a range of other nonpregnancy-related motivators for behavior change [17]. Regardless of women’s pregnancy intentions, optimizing health behaviors in the preconception period can improve every woman’s health and, in the event of pregnancy, can improve the health of future generations.

Digital health is defined by the World Health Organization as knowledge and practice associated with the development and use of digital technologies to improve health [20]. Digital health is facilitated by a range of existing technologies, such as mobile and online platforms and devices, as well as emerging data science, artificial intelligence, and robotics technologies [20,21]. A range of commercial online digital health resources have engaged women from the general population to make health behavior change, at least in the short term (eg, Michelle Bridges 12 Week Body Transformation [22], 28 by Sam Wood [23], Weight Watchers Digital [24]). However, cost is likely to be a barrier to access for some women. Online and social media platforms, such as Instagram and Facebook, are also highly influential in women’s decision-making around health behaviors [25]. Globally, 2.7 billion people use Facebook [26] and 1.0 billion use Instagram every month [27]. In Australia, approximately 3.5 hours are spent on online digital devices daily [28] and the average time spent exclusively on social media is 1.48 hours daily [29]. Modes of online engagement, and technology to support digital health resources, and ease of access are expanding rapidly [21]. With approximately 6 billion smartphone subscribers worldwide [30], opportunities to engage with almost any target market to promote behavior change have never been so numerous.

Digital health resources can promote behavior change [21,31], including in preconception populations. For example, Jack and colleagues [32,33] developed a virtual health professional to provide African American women with preconception health information and advice via computer and internet access. In a national randomized controlled trial in the United States, women in the intervention group were supported to address 50% of their identified preconception risks, significantly more than the control group [33]. This technology was tested in Australia with a multicultural sample of women aged 18-45 years [19,34]. Participants in this study, who all identified as being nonintenders, acknowledged the importance of health behaviors for preconception heath and considered the resource an acceptable way to provide preconception health information and advice. However, they ultimately were not motivated to engage with a digital health resource that promoted preconception health or pregnancy [19,34]. Participants were particular about what they liked and did not like. They called for digital health resources that are accessible on smartphones (the format tested with them via a computer), easy to navigate, and tailored to meet their individual preferences. Key considerations for those developing digital health interventions are the rapid evolution of information technology and possibilities in digital health [21], trends in user engagement, and keeping up with women’s preferences and expectations [19,34]. Despite the opportunities afforded by digital health, the requirements for engaging and sustainable preconception health resources are not yet fully understood. This research aims to further explore how intender and nonintender women engage with digital health resources and other online platforms, including social media, to inform the design and development of a digital health resource (or resources) to support women, both intenders and nonintenders, to make positive behavior change in relation to their preconception health. This study reports on the first phase of a larger body of participatory design research to empower women to adopt and maintain healthy lifestyle behaviors that optimize their preconception health and overall health and well-being.

## Methods

### Study Design

**Figure 1:**
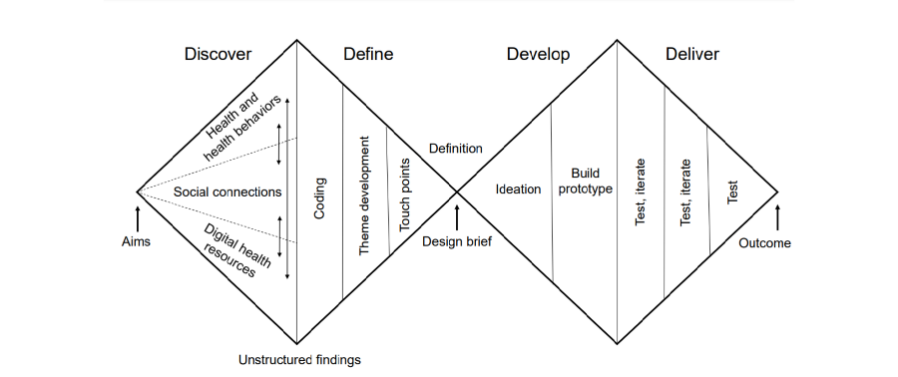
Double Diamond process, adapted for use in this research.

Our experience-based codesign approach involving women and community advisors was based on the Double Diamond process, an integrated design process that focuses on contextualization and explorative processes in phase 1 and ideation and development processes in phase 2 (Figure 1) [35]. Phase 1 is reported in this study, including a design brief with a range of considerations to scope the activities of phase 2. The anticipated output of phase 2 is a codesigned digital heath resource (or resources) that engages women with knowledge and support in relation to their preconception health behaviors. Two community advisory committees guided this research from the outset.

### Ethical Considerations

Ethics approval was obtained from the Monash University Human Research Ethics Committee (MUHREC reference: 28204). Participants provided informed consent via an online consent form. This full method is reported elsewhere (Walker et al, unpublished data, May 2022), and a summary is provided later.

### Setting, Participants, and Community Advisors

Study participants and community advisors lived in Victoria, Australia, and were aged 18-45 years. To be eligible, they needed to identify as being an intender (planning a pregnancy in the next 2 years or had a child in the past 2 years) or as being a nonintender (not planning a pregnancy in the next 2 years, with or without children already).

### Recruitment and Consent

Study participants and community advisors were recruited via advertisements sent to existing contacts at a range of women’s health groups in metropolitan Melbourne and regional Victoria, Australia, and via Facebook and Twitter. Community advisors were recruited and screened for eligibility in February and March 2021. Following the first community advisor meeting, participants were recruited and screened in April and May 2021.

### Data Collection

Community advisors were divided into intender and nonintenders groups based on whether they identified as being an intender or a nonintender. Four meetings for each group were held between March and October 2021. The meetings’ overall objectives were to introduce the study aims, gather feedback regarding the study design and results, and seek nonbiased guidance relating to how the interviews were conducted and phase 2 planning. The intender community advisors provided feedback in relation to the interviews being conducted with the intender participants, and the nonintender community advisors provided feedback in relation to the interviews being conducted with nonintender participants. These meetings were audio-recorded, transcribed, and added to the data set. Three in-depth interviews with each participant were conducted between June and September 2021. Interviews were designed to explore participants’ lived experiences in relation to their health and desired supports for healthy lifestyle behaviors. The first interview focused on participants’ perceptions of health and health behaviors, the second interview focused on social connections for health, and the third interview focused on digital health information and supports. COVID-19 restrictions meant that all meetings and interviews in this study had to be conducted online via Zoom. Meeting and interviews were audio-recorded using a Dictaphone, transcribed with the Descript transcription service, and then checked by 1 of the researchers. Community advisors and participants were de-identified with a unique participant number.

### Data Analysis

Data collection and analysis occurred concurrently. This was because data collected in previous interviews informed the subsequent interviews. This process involved rapid coding to gain insights from the first interview, which informed the activities designed for the subsequent interview with a degree of personalization based on the individual responses provided by the participants. Two researchers (authors SQ, a female research assistant and dietitian, and RW, a postdoctoral research fellow and dietitian, both with expertise in women’s health and qualitative research methods) double-coded a subset of the interview 1 transcripts to develop the initial coding framework. The remaining interview 1 transcripts were coded and cross-checked intermittently for consistency. The same process was used for interviews 2 and 3, with new codes being added to the initial framework. The transcripts from community advisor meetings were double-coded using the same framework. The community advisors discussed similar topics to those discussed in the interviews, creating a situation where the same coding framework used for the interviews could be used for the community advisor transcripts. NVivo software (QSR International) supported the analyses.

A deductive process was used for theme development. Codes were mapped to 3 focus areas of the in-depth interviews (perceptions of health and health behaviors, social connection, digital health information and support) and to the 3 components of the capability, opportunity, motivation, and behavior (COM-B) framework [36]. The 2 researchers developed summary statements for each intersection between the focus areas and the COM-B system before “touch points” were identified. Touch points in experience-based codesign are positive or negative experiences that elicit an emotional response [37]. In this study, we applied the term “touch point” to crucial aspects of the product or service design that must be present for participants to initially like it, have a desire or motivation to engage with it, actually engage with it, and then allow it to be a trusted and ongoing support for behavior change. For example, a woman may initially be drawn to a digital health resource because of its graphic design (eg, earthy tones, images of nature and well-being). She may like that the digital health resource has clear tabs for easy navigation and that the information provided is by a figure or person she relates to and trusts. Interacting with the resource may make her life easier, not harder. Touch points in this scenario may be labeled “calming,” “ease of use,” “relatability,” and “trust.” A degree of subjectivity may have been present in the identification of touch points for this study. Steps were taken to mitigate this by the 2 researchers (SQ and RW) discussing what touch points emerged as being most important to our participants with the rest of the research team. Repetition of some touch points across the intersections also indicated that they were important design considerations. The touch points generated the design brief that was scrutinized in the fourth round of community advisor meetings. Finally, themes and subthemes were derived from the summary statements and touch points.

## Results

### Community Advisor and Participant Characteristics

In total, 8 community advisors and 12 participants were included (Table 1), and 9 themes and 8 subthemes were derived from 124 codes, with overlap between themes (Table 2). Themes relating to digital health information and supports are reported here, while themes relating to health behaviors and social connections for health are reported elsewhere (Walker et al, unpublished data, May 2022).

**Table 1:** Community advisor and participant characteristics.

Characteristics	Community advisors (N=9)^a^	Participants (N=12)
Intender^b^, n (%)	3 (33.3)	6 (50.0)
Nonintender^c^, n (%)	6 (66.6)	6 (50.0)
Age (years), median (IQR)	29 (11)	31.5 (6)
Culturally and linguistically diverse, n (%)	4 (44.4)	3 (25.0)
Living with a disability^d^, n (%)	2 (22.2)	5 (41.7)
University educated, n (%)	8 (88.8)	10 (83.3)

^a^One of the community advisors only participated in 1 of the 4 group sessions.

^b^Intender: planning a pregnancy in the next 2 years or had a child in the past 2 years.

^c^Nonintender: not planning a pregnancy in the next 2 years, with or without children already.

^d^Posttraumatic stress, hearing impairment, mental health, severe endometriosis, attention deficit hyperactivity disorder.

**Table 2:** Summary statements, touch points, and themes (from participant and community advisor data).

		Perceptions of health and health behaviors^a^	Social connections for health^a^	Digital health information and supports
**Capability for health behaviors**				
	Summary statement	Capability comes from a holistic understanding of health, health information, and feeling of empowerment.	Social connections and shared experiences are integral to capability.	Digital health information and supports increase capabilities for health behaviors.
	Touch points	Health is valued. Trustworthy information.	Relationships with friends and family Shared experiences Being listened to	Trustworthy information Quality digital health information
	Theme(s)	Health is multidimensional, and a few key health behaviors are valued over all others.	Social connections and shared experiences build knowledge and increase confidence in decision-making.	Digital health information and support need to be evidence based and reputable to increase capability.
	Subtheme(s)	Health information can empower and disempower health behaviors. Everyday life can empower and disempower health behaviors.	Looking to other sources of information when health professionals are not trusted	N/A^b^
**Opportunity for health behaviors**				
	Summary statement	A range of resources create opportunities for health behaviors.	Social connections are shared experiences are integral to opportunity.	Digital health information and supports increase opportunities for health behaviors.
	Touch point(s)	Makes life easier	Relationships with friends and family Shared experiences Being listened to	Information and supports need to be timely and relevant. Online communities support social connections for health. Smartphone access is essential.
	Theme(s)	Personal responsibilities prioritized over health behaviors	Social connections and shared experiences increase opportunity for health behaviors.	Digital health information and support need to be relevant and increase opportunity for social connection.
	Subtheme(s)	Resources that make health behaviors easier	Being listened to shared experiences are important.	Ease of access and convenience are essential. Chatbots and virtual assistants may be useful but will not replace authentic social interactions.
**Motivation for health behaviors**				
	Summary statement	A desire to feel well and live well motivates health behaviors.	We thrive when we are together.	Digital health information and supports have the potential to increase motivation for health behaviors.
	Touch points	Pregnancy is a motivator when a pregnancy is planned or desired. Quality of life, including nature, rest, and relaxation.	We learn from each other. We support each other.	Clear and tailored information Variety and choice Privacy and trust Diversity and inclusion Aesthetically appealing Goal setting, tracking, and monitoring progress
	Theme(s)	A range of factors motivate health behaviors.	“We're not designed to be alone…We thrive when we're together.”	New digital health information and support need to be innovative to meet expectations of their target audience.
	Subtheme(s)	Pregnancy planning motivates behavior change.	A range of motivating benefits come from social connections.	New digital health information and support need to be innovative to meet expectations of their target audience.

^a^Reported elsewhere.

^b^N/A: not applicable.

#### Theme: Digital Health Information and Support Need to Be Evidence Based and Reputable to Increase Capability

Participants engaged with a range of digital health resources to increase their capability, including apps, online classes, podcasts, websites, and social media. They had clear expectations of the digital health resources they engaged with, including evidence-based information, tailored advice, and the presence of a moderator on online forums. Due to an abundance of online information and “a lot of misinformation out there” (participant 3, nonintender), participants were aware of the importance of health literacy and discerning between trustworthy and unreliable information. Google was a starting point for health information searches, along with recommendations from social connections, online reviews, and a number of downloads. Participants wanted to know that other people respected and valued the information they were accessing.

In terms of resources…I did a lot of Googling. I would look up what's good, what's happening through the pregnancy cycle and what's good to be, you know, to be done during pregnancy…Um, so I did look up a few resources…I think specific applications like “Baby Centre” has some good resources, even the NHS Pregnancy Guide [National Health Service, UK].

Participant 6, intender

#### Theme: Digital Health Information and Supports Need to Be Relevant and Increase Opportunity for Social Connection

Digital health information and support on social media (mostly Facebook and Instagram) increased the participants’ opportunity to search for information, access online communities, and learn from others who had similar experiences to them. Online communities also facilitated opportunities for social connections that were considered of utmost importance to our participants. Despite many participants and community advisors being only passively involved in online discussions (eg, not posting and only reading) or engaging intermittently, these forums seemed to be highly valued.

I think for me, even though I don't post or comment, I like to be able to read other people, uh, their posts, uh, and the advice that other people provide…Yeah. So that's, that's my personality for someone who is like an introvert and doesn't talk…But it [online communities] also caters to someone who's an extrovert and likes to really ask questions and ask opinions and provide advice.

Community advisor 1

Participants who valued online communities tended to be those most likely to experience barriers to meeting with people face to face (eg, had a chronic condition, caring for small children) or were at a time in life when their need for support was higher (eg, pregnancy).

I would definitely put an online community in my inner circle. When I fell pregnant with my oldest one, he's 6 now…I was holding on to the “What to expect when you are expecting” App…Like a lifeline, because I was terrified of what was going on. I was curious, I was all those things nervous, excited.

Community advisor 2; the term “inner circle” was used during the interviews to define those who participants relied on and trusted most.

It's [online community] somewhere between inner circle and community, to be honest. Um, I feel like obviously a lot of [online community’s] following it's my inner and broader community anyway. Um, but I feel like because we're discussing such vulnerable things and, um, people are coming back with responses saying, I understand, and I feel this, and I've been through this and everyone kind of feeling so comfortable and nurtured in that space that…I'm having conversations publicly on there that I wasn't having privately 2 years ago in my inner circle, which is really weird. But it's such a beautiful space, um, that I feel like that's kind of somewhere between inner and community.

Participant 3, nonintender

##### Subtheme: Ease of Access and Convenience Are Essential

Factors that increased the participants’ opportunity to use digital health information and support were access to hardware (eg, smartphones, tablets, and computers) and cost. Participants mostly used their smartphones and were happy to pay for health apps and online programs if they were perceived to be valuable.

I tend to use the mobile phone a lot more. Um, like, you know, when I'm kind of done with work, um, I use the phone a lot more.

Participant 6, intender

Like, I'm quite happy to pay for the, for apps or information if, if it was laid out to start with…

Participant 5, intender

##### Subtheme: Chatbots and Virtual Assistants May Be Useful but Will Not Replace Authentic Social Interactions

Participants were asked about their experiences with chatbots and virtual assistants. Most had interacted with them for practical, non-health-related activities, such as booking appointments or product queries. Most reported that they would be willing to interact with a chatbot or virtual assistant for health but described potential limitations. If participants used a chatbot or virtual assistant for health, they would need to trust they were receiving expert advice. Participants were less likely to interact with a chatbot or virtual assistant if it was not a person they were interacting with, if they did not receive timely responses, or if responses were not tailored to meet their needs.

I think you can tell when it [chatbot] is not a person…So I think that particularly from a health point of view, it maybe, sort of, needs to be very clear that’s what it is. That it's not, there's not a person at the end of this chat. This is just a direction tool or something…If it was a robot, I don't think I’d use it.

Participant 2, intender

#### Theme: New Digital Health Information and Support Need to Be Innovative to Meet Expectations of Their Target Audience

Participants had clear expectations of digital health information and supports they wanted. When asked about desirable features that would to motivate them to engage, the participants requested (1) resources with a clear purpose; (2) resources tailored to their needs; (3) privacy and opportunities to participate anonymously; (4) trust, openness, and honesty from other participants (fostering a sense of community); (5) diversity and inclusion; and (6) functionality that facilitates goal setting and data tracking to monitor their progress. Digital health resources also need to be visually appealing, easy to navigate, and accessible for those with hearing and visually impairments and should offer a variety of ways to engage (eg, Facebook groups, Instagram, Podcasts, and moderated forums).

I wouldn't want one resource to be all of general health, if that makes sense. Like, I would prefer it to be multiple or that resource to be more specific to women's health; or even that's very broad. Like reproductive health, for example, what would be helpful as a smaller area of the topic.

Participant 5, intender

One of the successful things I think of these groups is that there is those multiple components. Like, you have the podcast and you have the community. Um, I don't know if either would be so successful without the other.

Community advisor 3

In relation to preconception health, participants identified that tracking from preconception to pregnancy and birth would be helpful.

It would be cool to continue into pregnancy…So like especially spending money on an app. You don't really want to be like, “oh man, now I'm pregnant. Now I need a new app.” It would be good to continue on the same thing that you can still be tracking…

Participant 4, intender

Due to COVID-19 lockdowns, participants were working from home on their computers. The participants expressed wanting to get away from their computer screens at the end of the work day. If they were to engage with a digital health resource, they wanted to use one that seemed to be recreational.

Like it's, unfortunately it's [being online] just part of your whole day really…I just saw something on Twitter before…One of the people who has done some work with us is like, “Is anybody else just not interested in having Zoom parties?” Because by the end of a work day, I just, last thing I want to do is look at a screen…

Participant 2, intender

Participants created a huge list of digital health information and supports that they had accessed. Some of this information and support was developed by governments (Better Health Channel, National Health Service [NHS], United Kingdom) and large organizations (Baby Centre, Royal Women’s Hospital). Some of this information was developed to meet a perceived need and for commercial purposes (Keep it Cleaner, Chemical Maze, Noom), while some information and support were developed because of an unmet need and not for commercial purposes (Private Parts, Facebook groups). Participants did not list any resources that had been solely developed by health professionals or academics. In fact, some of their comments cautioned against this.

To me, um, it would need to be coming from like someone [a face or advocate] who opens the story. Like, “Hey, I'm 30 years old. I don't think I'm going to have kids. This is my situation.” Um, I think that it would be quite interesting…Like, as SP was saying, like, um, there might be online forums out there, but you always search for someone who is in a very similar situation to you…I think that's why “She's on the Money” has been successful because it's run by young woman. Um, so, yeah. It just like, it cuts through a little bit more…Rather than like, “Hey, we're like, you know, from our Ivory Tower or institution…” in the messaging. Yeah. You know what I mean?

Community advisor 4

Our community advisors also made some relevant comments in relation to the overall aims of this project to motivate women to engage with digital health resources for health behavior change.

I think that, that organic, um, like that organic genesis I think is really quite an important part. I think in terms of trust…I just like, I think that I've got a couple of friends who are involved with, um, a big breastfeeding group on Facebook called the Breastfeeding Cooperative…I think it started quite small and it's grown to be like, you know, over a hundred thousand members. And, um, you know, they've got this huge admin team now and it's massive. Um, and a lot of the women who moderate the group are doctors or nurses or like are professionals in some way, but it, it did happen very organically…It's a group that is really trusted among a lot of Australian women. But I think if it had started from like a health promotion, like deliberate, like “Let's, let's make a health promotion intervention!” I don't think it would have worked…

Community advisor 5

What does a health promotion intervention that women trust and rely on and engage with look like? I suppose it looks like something that you want to be a part of. Like, you want to actually be socially involved because you get something out of it…I like to feel how I give something back to that same community. And that's what makes it community rather than a classroom…I suppose, in order for them to be successful, I feel like there has to be a bit of learning, but also a bit of teaching taken on by everyone…I think you, you feel connected to them because of that shared experience. So if you create something that could generate that, that information-giving along with that social connectedness…I think you'd have a far greater buy-in from a lot of people.

Community advisor 2

Our discussions with the community advisors and participants identified themes and touch points (Table 2) that informed the development of a design brief to inform phase 2 of this research (Multimedia Appendix 1).

## Discussion

### Principal Findings

Our pregnancy intender and nonintender participants were already engaging with a range of digital health resources, including social media, to optimize their preconception health, general health, and overall well-being. They actively sought health information that they considered to be evidence based, trustworthy, and relevant. Desirable attributes of digital health resources were related to convenience. Our participants were busy and wanted information and supports that made their lives easier. Online interactions that facilitated meaningful social connections for health were prioritized over simply having access to more health information. Our participants listed a range of preferences for designers to consider as they leverage off information technology, the internet, and social media to develop digital health resources that promote preconception health behaviors.

### Limitations

This research had equal representation from pregnancy intender and nonintender participants who were interviewed 3 times over 3 months. This longer period of engagement allowed interviewers to delve more deeply into the participants’ lived experiences, their health behaviors, and supports for health. Ongoing interaction over 3 months also facilitated trust and rapport between participants and the interviewers. Additionally, ongoing data analyses enabled the findings from one interview to inform the next interview, and interviews were tailored to individual participants. The age range of participants was a limitation of this study, with an absence of women aged from 18 to 26 years. However, this younger age group was represented in community advisor meetings, with 3 nonintender community advisors being aged less than 26 years.

Consideration needs to be given to the impact of COVID-19 in the interpretation of the results. Data collection occurred during 1 of several COVID-19 lockdowns in the state of Victoria, Australia, in 2021. The COVID-19 lockdowns impacted all health behaviors, including those considered most important by our participants (food, exercise, and social connection) [38]. Potentially, the value participants placed on these health behaviors may have been magnified. Social connection is protective at times of stress, and perceived social support can buffer the negative impacts of isolation and loneliness [39]. Again, this may have magnified the importance participants placed on being socially connected with others. The final interview that explored participants’ use of digital health information and supports occurred after many participants had been working from home or home-schooling children for approximately 18 months. This increased use of information technology for work and daily tasks may also have altered how some participants described their use of digital health resources.

### Comparison With Prior Work

Digital health resources that promote health behaviors are plentiful, and women are engaging with them as it is expedient to them. This research highlighted women’s dependence on commercial or perhaps less formal digital health resources, including social media, such as Facebook and Instagram. Our participants valued the opportunities these platforms created for sharing experiences and learning from others (Walker et al, unpublished data, May 2022). Individuals are increasingly turning to experience-based information and support as opposed to expertise-based information and support provided by health professionals [40]. This challenges how health promoters and designers approach the development of digital health resources that are competitive in this space [41].

Examples of the successful integration of health promotion with social media platforms can be found in the use of existing online communication platforms, such as Facebook, WhatsApp, and free online learning platforms designed specifically for laypeople [42,43]. For example, the *FeedFinder* mobile phone app supports breastfeeding women to identify, review, and share information around public breastfeeding spaces [44,45]. This digital health resource was developed in consultation with women by a multidisciplinary research team from Newcastle University, United Kingdom, highlighting the importance of user involvement in design and how some digital health interventions are more effective when targeted to communities where information is shared [44]. Another example is the use of Massive Open Online Courses (MOOCs) for learning and discussion regarding a range of topics, including health [46-48]. Developed by Monash University, Australia, the MOOC Food as Medicine provided learners with education around nutrition as well as a moderated question and discussion forum [43]. Between May 2016 and February 2018, the course attracted >81,000 learners [41]. The evaluation found that learners were open to nonexpert advice from other learners because this feedback was timely, easily accessible, and often with personal anecdotes [41]. A common feature of these examples with our research is the value users placed on opportunities to interact and learn from each other. Experience-based information and support were preferred over expertise-based information by our participants. This reinforces the need to consider the strengthening influence of online voices and the importance of sharing experiences when developing digital health resources for contemporary women [40].

Future resources to promote behavior change, digital or other, should be codesigned with target audiences to ensure they meet their specific needs and preferences and promote ongoing engagement [49]. Our particular audience targeted in this research was broad. Essentially, a public health view of the preconception period includes all reproductive-age women [2,4]. We noted more similarities than differences between our intender and nonintender participants. Social connections, including being listened to and hearing from the shared experiences of others, were paramount for all our participants’ health and health behaviors (Walker et al, unpublished data, May 2022). In relation to digital health resources, participants primarily wanted resources that were easy to navigate and made their lives easier. This was confirmed with the feedback and oversight of our intender and nonintender community advisors who agreed that social connections should be integral to planning in phase 2. A systematic review of user engagement with digital health resources for mental health also found that social connections and customized content are enablers for engagement, while technical and navigation difficulties are a barrier [50]. The other touch points we identified, including privacy and the importance of images that represent quality of life (eg, nature, rest, relaxation), were incorporated into a design brief format and shared with our community advisors for feedback. Slight but important alterations were made to the design brief after the final community advisor meeting, demonstrating the importance of community engagement throughout the research process to ensure that participant and community voices are represented accurately [51].

### Conclusion

Women engage with digital health resources and other online platforms, including social media, to support their health behaviors and overall health and well-being. Women have clear expectations of what they want from digital health resources. These should be evidence based, reputable, and enable ease of access to fit in with busy lives. Social connections are paramount for women. Therefore, designers of digital health resources should consider how they can increase opportunities for women to connect and learn from each other to promote health behaviors. Codesigning digital health resources is important to successfully engage women with digital health information and support to increase their capability and create opportunities and motivation to support positive health behaviors.

## Appendix

Multimedia Appendix 1 Design brief to inform phase 2 of this study.

## Abbreviations

COM-B: capability, opportunity, motivation, and behavior
MOOC: Massive Open Online Course
