# Supplementary material for: Understanding Preconception Women’s Needs and Preferences for Digital Health Resources: Qualitative Study
Source: JMIR Form Res. 2022 Aug 5;6(8):e39280. doi: 10.2196/39280 (PMC9391970; doi:10.2196/39280)
Supplement: Multimedia Appendix 1 [file formative_v6i8e39280_app1.docx]

**
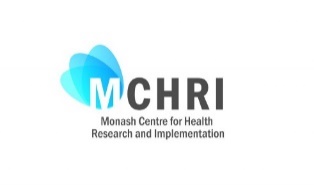
**
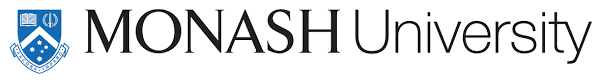
 **
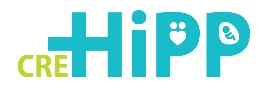
**

**Monash Centre for Health Research and Implementation + Action Lab**

**DESIGN BRIEF**

Project title: Supporting women with behaviour change for preconception health.

Project developer: Action Lab, Monash University.
Project manager: Jue Xie.
Email: jue.xie@monash.edu

**Project purpose and opportunity:** Women’s preconception health impacts on fertility, pregnancy outcomes, infant health and the future health and wellbeing of mothers and their children. Preconception health promotion tends to focus on women who are actively planning to become pregnant and the critical weeks around conception. Ideally, women should be supported to adopt and maintain health behaviours months and even years before conception. Importantly, aiming for optimal health regardless of current pregnancy intentions can improve the health of women and their babies where pregnancy is unplanned, and improve overall health and well-being of women who never have a pregnancy.

**Context and target audience:** From a life-course and market segmentation perspective, women may be classified as pregnancy ‘intenders’ (critical weeks around conception or actively planning to become pregnant) and pregnancy ‘non-intenders’ (when it is possible for a woman to become pregnant and her health behaviours have the potential to impact on pregnancy outcomes but she is not planning a pregnancy). These women are in their reproductive years. Considering that adolescents have very specific needs in relation to the sexual and reproductive health, this project targets women aged 18 – 45 years, both intenders and non-intenders. (Note: Our Community Advisory Committee suggested that this broad age range could be an issue at our fourth meeting, October 25^th^ 2021. This was after being presented with the results.)

**Design problem:** Community awareness of the importance of preconception health is low. Many women only seek preconception care if they do not become pregnant after trying. A plethora of interventions targeting pregnancy intenders have been developed and evaluated by health professionals and researchers but many of these interventions are yet to be translated into real world settings. Interventions specifically targeting non-intenders are harder to identify as these women do not resonate with the term preconception. Overall, public health messages have not engaged intenders or non-intenders regarding the importance of preconception health or promoted positive health behaviours. On the other hand, a range of engaging online resources and movements have developed organically through social enterprises and networking. These resources and movements have empowered many women to improve various aspects of their health.

**Objective:** To empower women to adopt and maintain healthy lifestyle behaviours that optimise their preconception health and overall health and wellbeing.

**Messages (key ideas to be considered in ideation):**

- A holistic view of health (physical, social, emotional).
- Nutrition, sleep and exercise are considered important health behaviours.
- Achieving health goals and positive physical and emotional health outcomes motivates continuation of health behaviours.
- Planning a pregnancy motivates health behaviours.
- Future pregnancy does not motivate health behaviours for those not planning a pregnancy.
- Social connections have the biggest impact on health (physical, social, emotional) and health behaviours (in terms of capability, opportunity and motivation).
- “We're not designed to be alone… We thrive when we're together.” (P02 Intender).
- Family and friends considered most important social connection.
- Shared experiences build knowledge and increase confidence in decision-making.
- Being listened to has a positive impact on confidence and feelings of empowerment.
- Online communities facilitated social connections; for many, participation was passive.

**Attitude (style and tone to be considered in ideation):**

- Women are disempowered by too much information and/or not being listened to.
- Outdoors, nature.
- Natural therapies accessed when there are difficulties with preconception.
- Women suffer from chronic illness, are busy, put their children’s needs before their own, and are living in a pandemic. Therefore, resources and support need to make life easier.
- Evidence-based but mindful of power in-balances between health professionals/academics and everyday women.
- Messages from ‘peers’ or someone who understands/relates to women.
- Not all women are looking to have children – design should be mindful of this.
- Messaging should be around supporting all women (whether they are an intender or non-intender) to live healthy lives and achieve optimal wellbeing.

**Deliverables and format:**

- Digital health information, online resources or social networks that are excellent and meet high expectations.
- A variety of ways to engage including health apps, online classes, podcasts, websites and social media.
- Desirable features: a clear purpose, tailored to meet needs, privacy, trust, openness, honesty, diversity – considers the needs of a range of backgrounds, inclusion – particularly with language, functionality that facilitates goal-setting and feedback on progress, practical and easy “bite-sized”, “short and sweet”.
- Visually appealing.
- Easy to navigate.
- Accessible for the hearing and visually impaired.
- Be separate from industry or profit making, or at least be mindful of the influence of industry.

**Timeline:** TBC – Action Lab.

**Budget:** TBC – Action Lab.
